# Surgical management of compensatory sweating: A systematic review

**DOI:** 10.3389/fsurg.2023.1160827

**Published:** 2023-03-22

**Authors:** Domenico Loizzi, Diletta Mongiello, Maria Teresa Bevilacqua, Federico Raveglia, Alfonso Fiorelli, Maria Teresa Congedo, Nicoletta Pia Ardò, Francesco Sollitto

**Affiliations:** ^1^ Department of Medical and Surgical Science, University of Foggia, Foggia, Italy; ^2^ Department of Thoracic Surgery, San Gerardo Hospital, Monza, Italy; ^3^ Department of Translation Medicine, Thoracic Surgery Unit, Università della Campania “Luigi Vanvitelli”, Naples, Italy; ^4^ Dipartimento di Medicina e Chirurgia Traslazionale, Università Cattolica del Sacro Cuore, Roma, Italy

**Keywords:** compensatory sweating (CS), compensatory hyperhidrosis (CH), unclipping, diffuse sympathectomy, sympathetic nerve reconstruction

## Abstract

Endoscopic thoracic sympathectomy (ETS) surgery is a highly effective treatment of primary hyperhidrosis (PH) for the palms, face, axillae. Compensatory sweating (CS) is the most common and feared side effect of thoracic sympathectomy. CS is a phenomenon characterized by increased sweating in sites distal to the level of sympathectomy. Compensatory sweating is the main problem for which many patients give up surgery, losing the chance to solve their problem and accepting a poor quality of life. There are still no treatments that offer reliable solutions for compensatory sweating. The treatments proposed in the literature are scarce, with low case histories, and with uncertain results. Factors associated with CS are extension of manipulation of the sympathetic chain, level of sympathetic denervation, and body mass index. Therapeutic options include non surgical treatment and surgical treatment. Non surgical treatments include topical agents, botulinum toxin, systemic anticholinergics, iontophoresis. Surgical treatments include clip removal, extended sympathectomy and sympathetic chain reconstruction, although the efficacy is not well-established for all the methods. In this review we provide an overview of the treatments and outcomes described in the literature for the management of compensatory CS, with focus on surgical treatment.

## Introduction

Endoscopic thoracic sympathectomy (ETS) is the standard surgical treatment for palmar, facial and axillary hyperhidrosis with a success rate greater than 95% (1).

Compensatory sweating (CS) is the most common and feared side effect of thoracic sympathectomy and is a phenomenon characterized by increased sweating in sites distal to the level of sympathectomy. CS can range from mild to severe, with a percentage that can reach 98% depending on the case (2).

Patients with intense symptoms of CS feel such discomfort that they regret surgery; they have to change their clothes several times a day. This symptom greatly affects daily and professional activities and has severe consequences for patients’ quality of life.

The treatments for CS range from lifestyle control, to pharmacological, topical or systemic treatments, iontophoresis, up to surgical treatments for severe forms.

For lifestyle control we can consider weight control, non thermogenic diet, regular physical activity. Pharmacological treatments include topical agents, botulinum toxin injections, and systemic anticholinergics.

Patient choices are influenced by the severity of the side effect and the patient's compliance with the various treatments offered. Some treatments are minimally invasive, but must be performed for a long time, if not for life, while others are more invasive but have a longer duration or are expected to be definitive (2).

Several surgical techniques for CS have been proposed depending on the previous sympathetic surgery. These techniques can be grouped into three categories: (a) unclipping; (b) extended sympathectomy/sympathicotomy; (c) sympathetic nerve reconstruction.

In this review we focus on the evidence actually available in the literature on these techniques.

## Materials and methods

### Search strategy

In October 2022 we conducted an extensive and systematic literature search to identify all relevant studies published up to October 2022.

The following databases were searched: Pubmed, Scopus, Google Scholar.

The Keywords used were compensatory sweating, compensatory hyperhidrosis, reflex sweating, reflex hyperhidrosis.

### Selection of studies and data collection

The selection of studies was performed by two reviewers. After excluding duplicate studies, the titles and abstracts were analyzed.

We analyzed in their entirety the papers where a keywords was in the main topic or was included in the title or abstract.

### Inclusion criteria

We selected studies that met all of the following inclusion criteria: (1) studies published in English; (2) studies involving series of at least 5 patients undergoing surgical treatment for compensatory hyperhidrosis following ETS with patient-reported outcome; (3) studies reporting the number of patients with improvement on the total number of patients treated and with documented results; (4) the most recent study in case of duplication of data of the same author.

### Data extraction

Data extraction was performed by two reviewers, using a standardized Excel form. The number of patients with improvement out of the number of total patients treated and with documented self-reported outcome was reported in the tables as satisfaction rate.

Data regarding sympathetic levels treated in the previous surgery were summarized in the tables in 3 categories: T2 and below, T3 and below, T4 and below.

### Subgroup analysis

Patients treated with clips removal, reconstruction surgery or diffuse sympathectomy were separately grouped.

## Results

After excluding duplicate publications, there were 4,976 studies. After analysis of the titles and the abstracts, we selected 54 studies for full analysis.

After the full text analysis, 32 studies were excluded because they were not relevant (*n* = 21), or they were case reports or with case histories less than 5 (*n* = 7), or they did not report outcomes described in the inclusion criteria (*n* = 4).

We selected 22 studies relevant for the analysys, of which 14 met the inclusion criteria for quantitative synthesis and were included in tables.

Table 1 shows the baseline characteristics of the selected studies: author, origin, year of publication, techniques for reversal (grouped in unclipping, diffusesympathectomy and nerve reconstruction), number of patients treated with documented results, kind of outcome of the reversal surgery in the study and the level of previous surgery (T2/T3/T4 and below).

**Table 1:** Characteristics of the studies.

Author	Origin	Year	Techniques for reversal	N patients treated, with documented results	Kind of outcome of the reversal surgery in the study	Previous surgery		
						T2 and below	T3 and below	T4 and below
Lin (3)	China	1998	Clip removal	5	Secondary	5		
Reisfeld (4)	USA	2006	Clip removal	25	Secondary	22	3	
Chou (5)	China	2006	Clip removal	13	Secondary	nd	nd	nd
Jo (6)	South Korea	2007	Clip removal	9	Secondary	3	6	
Kang (7)	South Korea	2008	Clip removal	14	Primary	12	2	
Sugimura (8)	Canada	2009	Clip removal	31	Primary	26	5	
Hynes (9)	USA	2015	Clip removal	8	Primary	5	3	
Kara (10)	Turkey	2019	Clip removal	8	Secondary	5	3	
Yamamoto (11)	Japan	2019	Diffuse sympathicotomy	8	Primary	2	5	1
Moon (12)	South Korea	2020	Diffuse sympathicotomy	44	Primary	13	25	6
Vasconcelos (13)	Brasil	2020	Diffuse sympathicotomy	12	Primary		12	
Haam (14)	South Korea	2010	Nerve reconstruction	17	Primary	12	5	
Rantanen (15)	Finland	2016	Nerve reconstruction	19	Primary	8	11	
Gebitekin (16)	Turkey	2021	Nerve reconstruction	15	Primary	nd	nd	nd

Table 2 shows the outcomes of the reversal surgery with the satisfaction rate, time to reversal (range in months) and follow-up declared from reversal (range in months).

**Table 2:** Outcomes of reversal surgery.

Author	Year	Techniques for reversal	Satisfaction rate^a^ (%)	Time to reversal (range in months)	F-up declared from reversal (range in months)
Lin (3)	1998	Clip removal	4/5 (80)	1–2	6–8
Reisfeld (4)	2006	Clip removal	16/25 (64)	nd	nd
Chou (5)	2006	Clip removal	10/13 (77)	nd	nd
Jo (6)	2007	Clip removal	8/9 (89)	1–2	6–30
Kang (7)	2008	Clip removal	9/14 (64)	1–2	6–65
Sugimura (8)	2009	Clip removal	15/31 (48.4)	1–57	1–72
Hynes (9)	2015	Clip removal	5/8 (62.5)	3–70	1–54
Kara (10)	2019	Clip removal	2/8 (25)	1–30	10–31
Yamamoto (11)	2019	Diffuse sympathicotomy	8/8 (100)	>6	14–22
Moon (12)	2020	Diffuse sympathicotomy	20/44 (45)	1–24	8–29
Vasconcelos (13)	2020	Diffuse sympathicotomy	8/12 (66.6)	nd	nd
Haam (14)	2010	Nerve reconstruction	9/17 (53)	4–111	1–45
Rantanen (15)	2016	Nerve reconstruction	14/19 (73.6)	6–144	6–180
Gebitekin (16)	2021	Nerve reconstruction	14/15 (93.3)	7–96	nd

^a^ Satisfaction rate is described as the number of patients with improvement out of the number of total patients with documented outcome, expressed as fraction and percentage.

## Discussion

The pathophysiological mechanism by which CS develops remains unknown to date. The risk of developing compensatory hyperhidrosis is influenced by many variables. If we consider all forms of compensatory hyperhidrosis, from the lightest to the most severe, the percentage of subjects that is affected in the literature can reach up to 98% (2, 17).

In 2006, Chou (5) suggests that changes in sweating patterns after sympathetic surgery may be attributable to a reflex response in the sweating center of the hypothalamus, and not at all to a compensatory mechanism. For this reason he suggests using the term “reflex sweating” instead of “compensatory hyperhidrosis”. The distribution of sweating control pathways may show some variability in the population.

This could explain why the same surgery for primary hyperhidrosis (PH) could have different outcomes in different patients.

Several authors have pointed out that surgery for the craniofacial hyperhidrosis, therefore surgery on T2, increases the risk of CS (17). Sympathectomy at the T2 level probably causes interruption of the negative feedback to the hypothalamus and this seems to be shown to be the district most at risk for the development of CH (2, 18).

Some research suggests that CS is associated with extensive sympathicotomy, while others report that the extent of sympathicotomy has no association with the degree of CS.

The poor knowledge of the mechanisms underlying CS are reflected in the heterogeneity of treatments currently proposed for its treatment.

The literature actually available on the surgical treatment of compensatory hyperhidrosis is scarce and with small case series. The outcome's evaluation is not well established. The most common methods to evaluate surgery are scales of patient- reported outcome, with different questionnaires administered by different authors.

Furthermore, patients who only want to solve the CS, maintaining the benefits obtained with the first intervention, must be distinguished from patients who, in addition to the CS, regret the excessive dryness of the hands.

The objective of reversal surgery could be an attempt to return to the conditions before surgery, or an extension of the action of the surgery on the body areas affected by CS. Patients with primary palmar hyperhidrosis are more likely to have mild or moderate mental disorders, and postoperative compensatory sweating may impact the satisfaction of surgery. In addition, the personality characteristics of patients are related to compensatory sweating (19). For this reason it is recommended that all patients should take psychological states evaluation before any kind of sympathetic surgery.

Reversal when the chain has been cut is challenging, whereas reversal when the chain has been clipped is straightforward.

The most documented technique is clip removal, and it can be performed only in patients submitted to clip placement. In our review we describe 8 series of clip removal (Tables 1, 2) starting 1998, with a total of 113 patients treated and with documented results. Out of these, 69 (61%) patients were satisfied with the result of the procedure. The treatment of compensatory sweating after clipping and the evaluation of its effectiveness was described in secondary outcomes in 5 studies while it was primary outcome in the remaining 3 studies. In these series the satisfaction rate ranges from 25% to 89%, without a clear trend over the time.

The hypothesis of the regeneration of the sympathetic chain after clip removal is controversial. Some authors suggest that clip removal time, reported as the time between the placement of clips and their removal, may be a variable affecting the outcome (9, 10). In our data the differences in the time to reversal can therefore partially explain the differences in the results (satisfaction rate).

However some authors think that degenerative and irreversible changes occur at the level of the sympathetic nervous system following the placement of clips even if removed after a few days.

In a study of 2012 on a swine model, performing clipping, unclipping and extirpation with pathological examination, the authors observed Wallerian degeneration as early as 10 days after clip placement. They conclude that clipping cannot be considered a reversible technique (20).

In another 2014 animal model study, however, 12 weeks after unclipping, severe histological damage in the sympathetic trunk had clearly decreased, which suggests in theory that application of metal clips to the sympathetic chain is a reversible procedure if only the observation period is prolonged (21).

Other variables that can influence the results of the clipping technique are the degree of compression exerted by the clips and the differences between the clips themselves. For example, clips can be single-branch or dual-branch.

The satisfaction rate shows big variability as shown in Table 2.

In the various studies, the time to reversal can also be a variable that influences the result. In the selected studies, the time to reversal, where specified, ranges from 1 to over 70 months depending on the cases.

Regarding the results, the largest series found in literature is by Sugimura et al. reporting data about 31 patients undergoing unclipping in a reversal time period between 1 and 57 months and with a satisfaction rate of 48.4% (15/31). They conclude that reversal by unclipping offers acceptable results and should be considered in selected patients (8).

The best result as satisfaction rate after unclipping is reported by Jo et al. who shows 8/9 patients (89%) satisfied by the reversal surgery. They presented a new protocol for clip removal under local anesthesia. During the first surgery they place a suture between the tip and the body of the clip applicator that is fixed in the subcutaneous tissue; during reversal the clip is easily detected and removed with being pulled back (6).

From currently available data the reversibility of clipping remains controversial. The number of individual cases is minimal (5–31 patients).

The consensus of the International Society of Sympathetic Surgery states that unclipping has a placebo effect (10).

The issue about the reversibility of effects of sympathetic clipping remains empirical.

If there is little data on clip removal, even fewer are those concerning diffuse sympathectomy.

An important advantage of this technique is that it can be performed after any type of ETS already performed. It consists in the extension of the surgery on ganglia, usually at a lower level, with the aim of disrupting the activity of the ganglia responsible for the CS.

In our review only 3 studies met the inclusion criteria. These studies were published in 2019–20 and collected 64 patients. Satisfaction rate ranges from 45% to 100%. The study declaring a satisfaction rate of 100% seems to be promising, even if it involves only 8 patients (11).

In this report the authors described an original technique: patients with severe CS were treated by observing blood perfusion of the skin with laser speckle flowgraphy (LSFG), stimulating each sympathetic nerve and ganglion with an electrosurgical unit. LSFG allowed the exact identification of the ganglia corresponding to the CS areas. After identification of the ganglia responsible for the CS, ganglionectomy was performed.

In the remaining studies sympathectomy was applied to the lower sympathetic chain starting from R5 to R8 or from R5 to R11, eventually associated with lumbar sympathectomy L3 in case of severe plantar hyperhidrosis. In these studies the sympathectomy was performed without intraoperative monitoring of blood perfusion for determining the connections between ganglia and skin areas affected by CS. In these 2 studies the satisfaction rate was 45% for Moon (12) and 67% for Vasconcelos (13).

The latter concludes that extended R5–R8 thoracic sympathectomy for compensatory hyperhidrosis seems to be an effective and safe alternative to the other techniques with promising results.

Extended sympathectomy is feasible, but laser fluoroscopy equipment described by Yamamoto is not commonly available in institutions.

The efficacy of a diffuse sympathectomy can also be considered for preventive purposes, performing it directly during the first operation for sympathectomy for hyperhidrosis (17).

Han et al. designed a new sympathectomy method to prevent severe CS by expanding sympathectomy as low as possible beyond R8, even to R12. Their results showed a significantly reduced degree of CS and there were no severe CS cases without major complications (22).

Sympathetic nerve reconstruction (SNR) is a complex surgical procedure. Reports on sympathetic nerve reconstruction are also scarce. Three papers met the inclusion criteria, with a total number of 51 patients treated with documented results.

Of these, 37 (72.5%) patients were satisfied after the procedure.

Proximal and distal ends of the previously resected sympathetic chain are exposed and cut. Either sural or intercostal nerve was used as a free graft and fibrin glue was applied to the contact surfaces. The nerve is generally anastomosed in the original direction.

The use of the intercostal nerve is preferable because it does not require additional surgical accesses, has more sympathetic nerve fibers, and can be used also as a pedicled graft harvested as a neurovascular bundle (14, 15); sural nerve can be used only as free graft and requires additional incision. Successful nerve anastomosis is generally obtained with fibrin sealant without suture technique. Rantanen and Telaranta report that approximately 75% (14/19) of their patients benefited from SNR after an average reversal time value of 87 month and that in 50% of these the improvement was significant; they conclude that SNR can be considered as a potential treatment option for patients with severe side effects from ETS which are unresponsive to conservative treatment (15).

This is a much more complex technique that requires more skills of the surgeon, more resources, and significantly longer operating times.

The results of this technique, considering the cost-benefit ratio, compared with other techniques do not actually seem to justify its use except in referral centres.

## Conclusion

Severe CS is a rare but hardly treatable complication of ETS. Candidates for ETS should be informed about risk factors for developing severe CS.

The experiences on the surgical treatment of CS seem to be still few.

CS has to be managed with lifestyle control, pharmacological treatment, and iontophoresis. Surgical treatment for CS should be offered to highly motivated patients in referral centers.

There is currently no evidence of a surgical treatment for CS recognized as completely effective. Informed consent must be clear about the real expectations of success of such treatments.
